# Health systems challenges, mitigation strategies and adaptations to maintain essential health services during the COVID-19 pandemic: learnings from the six geopolitical regions in Nigeria

**DOI:** 10.1186/s12913-024-11072-2

**Published:** 2024-05-14

**Authors:** Segun Bello, Rachel Neill, Ayodele S Jegede, Eniola A. Bamgboye, Mobolaji M. Salawu, Rotimi Felix Afolabi, Charles Nzelu, Ngozi Azodo, Anthony Adoghe, Munirat Ogunlayi, Saudatu Umma Yaradua, William Wang, Anne Liu, Olufunmilayo I. Fawole

**Affiliations:** 1 University of Ibadan, Ibadan, Nigeria; 2 The Global Financing Facility for Women, Children, and Adolescents, 1818 H ST NW, Washington, DC 204333 USA; 3 Nigeria Federal Ministry of Health, Federal Secretariat Complex, Phase III, Shehu Shagari Way, Central Business District, Abuja, Nigeria; 4 Gate Ventures, Seattle, Washington USA

**Keywords:** Health systems resilience, COVID-19, Adaption, Mitigation, Essential health services

## Abstract

**Background:**

The COVID-19 pandemic control strategies disrupted the smooth delivery of essential health services (EHS) globally. Limited evidence exists on the health systems lens approach to analyzing the challenges encountered in maintaining EHS during the COVID-19 pandemic. This study aimed to identify the health system challenges encountered and document the mitigation strategies and adaptations made across geopolitical zones (GPZs) in Nigeria.

**Methods:**

The national qualitative survey of key actors across the six GPZs in Nigeria involved ten states and the Federal Capital Territory (FCT) which were selected based on resilience, COVID-19 burden and security considerations. A pre-tested key informant guide was used to collect data on service utilization, changes in service utilization, reasons for changes in primary health centres’ (PHCs) service volumes, challenges experienced by health facilities in maintaining EHS, mitigation strategies implemented and adaptations to service delivery. Emerging sub-themes were categorized under the appropriate pillars of the health system.

**Results:**

A total of 22 respondents were interviewed. The challenges experienced in maintaining EHS cut across the pillars of the health systems including: Human resources shortage, shortages in the supply of personal protective equipments, fear of contracting COVID-19 among health workers misconception, ignorance, socio-cultural issues, lockdown/transportation and lack of equipment/waiting area (. The mitigation strategies included improved political will to fund health service projects, leading to improved accessibility, affordability, and supply of consumables. The health workforce was motivated by employing, redeploying, training, and incentivizing. Service delivery was reorganized by rescheduling appointments and prioritizing some EHS such as maternal and childcare. Sustainable systems adaptations included IPC and telehealth infrastructure, training and capacity building, virtual meetings and community groups set up for sensitization and engagement.

**Conclusion:**

The mitigation strategies and adaptations implemented were important contributors to EHS recovery especially in the high resilience LGAs and have implications for future epidemic preparedness plans.

**Supplementary Information:**

The online version contains supplementary material available at 10.1186/s12913-024-11072-2.

## Background

The COVID-19 pandemic remains the biggest global health systems shock of the 21st century leading to about 6.8 million deaths as of 26th February, 2023 [1]. The interventions implemented to control the pandemic have had far-reaching consequences, ranging from disruptions to socio-economic activities, to decline in health services provision and utilization. According to the World Health Organization (WHO), countries henceforth need to make trade-offs between the scale of direct response to health threats and the actions geared towards maintaining essential health service delivery, to mitigate the risk of system collapse [2]. 

Disruptions are defined as “unforeseen events that interfere with the provision of healthcare goods and services” [3]. During the COVID-19 pandemic, disruptions in health service delivery and decline in essential health services utilization was documented across all health systems including high, medium and low-income countries [4]. These disruptions were attributed to aspects of the COVID-19 pandemic response including lockdowns and reorganization of health service delivery with a shift in focus to COVID-19 control [2]. For example, in Europe, screening for cancers decreased by as much as 65 − 95% during the early phase of the pandemic [5]. In Africa, several health programmes including the malaria elimination programme, HIV/tuberculosis control, diabetes, and hypertension services were deprioritized during the pandemic [6, 7]. Heavy declines were also reported for maternal, child health and immunization programmes [8, 9]. effectively threatening the gains achieved in health programme outcomes over decades of investment [10]. These health programmes reported decline in service output as well as set- backs in performance indicators as similarly demonstrated during the West Africa Ebola outbreak pandemic. Analysis of the 2014–2015 Ebola outbreak suggested that the number of deaths caused by measles, malaria, HIV/AIDS and tuberculosis attributable to health systems failure during the Ebola outbreak exceeded deaths from Ebola [11–14]. 

The WHO health systems framework describes the core building blocks or pillars of the health systems which contribute to the resilience of a health system [15]. The performance of the health system in handling health crisis depends on its baseline capacity predating the crisis, as well as the magnitude of the crisis [2]. Kruk et al. defined health systems resilience as ‘the capacity of health actors, institutions, and populations to prepare for and effectively respond to crises; maintain core functions when a crisis hits; and, informed by lessons learned during the crisis, reorganize if conditions require it.’ [16] Thus, apart from maintaining the core functions of a health system, resilience includes the health system’s ability to transform, evolve and enhance its performance in improving the health of the population [17]. 

A well-prepared health system should have the capacity to maintain essential health services delivery to reduce morbidity and mortality from sources other than the cause of the health systems shock, throughout the duration of an emergency. Both demand and supply factors have been documented as challenges mitigating against the maintenance and utilization of essential services across health systems during the COVID-19 pandemic. The pandemic increased the workload for health systems, resulting in pressure and inadequate health workforce all over the world [5]. However, LMICs have been particularly affected from operating more vulnerable health systems with challenges that predated the COVID-19 pandemic. To compound the challenges of human resource shortages, about 50% of health facilities across Africa reported COVID-19 infection among staff, shortages in personal protective equipment (PPE), underfunding, reduced supply of medications and poor information systems [6, 18]. Most African countries are dependent on importation of essential medicines and products. These countries were affected by the disruptions in the global supply chain because drugs were not readily available or were expensive because of the high demand relative to supply [19]. Patients expressed difficulties in accessing medicines due to the high cost [20]. 

Geographic variability in the level of disruptions and restorations to EHS were reported within countries [21]. The COVID-19 high burden states/areas were likely to have experienced a higher level of restrictions and enforcements of protocols which could affect the levels of disruptions and the time taken for restorations. Furthermore, recovery may be slow, temporary, or partial depending on sub-national health systems resilience. Reported innovative adaptations to halt or reverse decline in EHS delivery included home delivery, use of phones, improved triaging, shift to remote consultations, and expansion of the scope of work of community health workers and task shifting [21, 22]. Limited evidence exists on the health systems lens approach to analyzing the challenges encountered in maintaining EHS delivery during the COVID-19 pandemic, particularly at the sub-national levels. Therefore, this study aimed to identify the health system challenges encountered during the COVID-19 pandemic and document the mitigation strategies and adaptations made across the geopolitical zones (GPZs) in Nigeria. The learnings will guide policymakers, decision makers and health administrators on how to improve health systems in Nigeria to ensure that they are resilient and prepared to respond to public health emergencies. Learnings from Nigeria especially on the mitigation and adaptation strategies may be transferrable to similar decentralized health systems.

## Methods

### Study setting

The study was qualitative in design involving interviews of key persons at state ministries of health (SMoH) and State Primary Health Care Development Agencies (SPHCDA) across the six geopolitical zones of Nigeria. Following the Alma Ata declaration in 1978, the primary health care (PHC) system became the fulcrum of health systems development in Nigeria. Not much progress was made in PHC however, until 1985 when the then Minister of Health adopted 52 Local Government Areas (LGAs) to build models based on the Alma Ata declaration [23]. Thereafter, the model was expanded to include all LGAs and the responsibility for overseeing the working of the PHC including immunization, antenatal care services was devolved to the LGAs [23]. PHC in Nigeria focuses on preventive services including immunization, antenatal care services, as well as the provision of basic health care services at the grass root level [23]. 

The Primary Health Care Under One Roof policy was introduced in 2010 and approved in 2011 [24]. It aims to strengthen the national health system by integrating all PHC services under one authority. By implications, all resources for PHC implementation are to be repositioned from all agencies, departments and ministries to the new State PHC development agencies or boards [24]. This initiative produced some improvements in health outcomes [25]. 

Like many other African countries, Nigeria has consistently failed to implement the 2001 Abuja declaration at which African heads of state pledged to allocate 15% of the annual national budget to health [26, 27]. Currently, the PHC system has deteriorated with most of the 30,000 PHC facilities across the country lacking the capacity to provide essential healthcare services thereby, transferring enormous pressure to the higher levels of healthcare [28]. The challenges PHC facilities experienced before COVID-19 included poor staffing, inadequate equipment, poor distribution of health workers, poor quality of healthcare services, poor condition of infrastructure, and lack of essential drug supply.

### Study design and approach

The study was part of a large national qualitative survey on resilience of the health system which aimed at identifying the key challenges to maintaining essential health services during the pandemic, from the perspective of subnational actors. This current report focused on the regional level data, but the other aspect of the study focused on comparative LGA-level data on how some LGAs overcame challenges and sustained essential health services, while comparable, neighbouring LGAs experienced ongoing disruptions [29]. 

### Study site and participants’ selection

The study enrolled subnational actors at the state level, across the six geopolitical zones of Nigeria. These participants were engaged in the COVID-19 response and were at decision-making levels such as directors, assistant directors and heads of programmes.

Selection of study sites was guided by multiple criteria namely High Resilience (HR) LGAs; COVID 19 disease burden and regional hotspots such as LGAs with the highest cumulative cases and those with international airport or land borders; security considerations, by avoiding LGAs with considerable security challenges such as LGAs with insurgents and banditry. The procedure for identifying high resilience LGAs has been described in detail elsewhere [29]. In brief, the general outpatient (GOPD) and Ante-natal care (ANC) health services data from the National Health Management Information System (NHMIS) (January 2019 – December 2021) was analyzed using the interrupted time series. The analysis identified HR LGAs. HR LGAs were defined as LGAs which experienced a recovery in service volumes within three months of decline precipitated by the COVID-19 pandemic. LGAs were then stratified and ranked within each geopolitical zones and, in combination with COVID-19 burden and security considerations, 12 h LGAs were finally selected from 10 states and the Federal Capital Territory (FCT) across the six geopolitical zones: South-West [3], South-South [2], South-East [2], North-West [1], North-Central [2], North-East [1].

Participants for the parent study were selected purposively across state, LGA, health facilities, and community levels. However, this report is limited to the analysis of response from state-level participants across the GPZs where two participants each were selected per state.

### Data collection

#### Data tool

A key informant guide was developed following extensive review of literature on health systems resilience and essential health service maintenance (see Supplementary File). The guide was pretested among similar personnel in Nasarawa state before data collection. The interview guide was sectioned according to: profile of the study participants; services used during COVID-19; data monitoring and use; self-regulation; adaptive-short term; integrated capacities or planning; relevance to maternal neonatal and child health (MNCH); and adaptive-long term. The data presented in this report focuses on the following sections of the tool: services used during the COVID-19 pandemic and self-regulation which contained information on participants’ assessment of changes in service utilization during the COVID-19 pandemic; reasons for the changes in Primary Health Centres (PHCs) attendance, challenges experienced by facilities to maintain routine services during the pandemic, specific countermeasures that the state governments took to overcome the challenges and activities done by the state governments to encourage clients to continue to utilize the PHCs.

### Procedures

The Federal Ministry of Health (FMoH) led the project and played an oversight role in data collection with supervisors leading research teams to the states. The interview teams paid advocacy visits to explain the purpose of the research and obtain the support of stakeholders in the ministries of health. The interview team comprised of a supervisor, a moderator and a note taker per state. All data collectors and supervisors were trained for the purpose of this research. All participants gave informed consent before they were interviewed face-to-face and audio-recorded in their offices. A data collection pause was implemented after the first few interviews during which the interviews conducted were transcribed, reviewed and feedback were communicated to the field teams. The interview took an average of 73 min. Data was collected between June through July, 2022.

### Data management

The recorded interview audios were transcribed verbatim in the original language of the interview. Transcripts were complemented with notes taken during the interviews. The transcripts, audio files and notes were labelled with unique identifiers that enabled data linkage across files. A data security protocol was implemented to safeguard against data breach. A Dropbox folder, which was only accessible to designated research team members, was created for the safe storage of the audio files, transcripts and summary notes.

Coding was done using *Atlas.ti.* One coder was involved in the coding the data while multiple coders coded subsets of the data for agreement. The entire research team interrogated the data and review the coding. Emerging sub-themes were categorized under the appropriate pillars of health system including (i) service delivery, (ii) health workforce, (iii) health information systems, (iv)medicines and supplies, (v) financing, (vi) leadership/governance [15]. 

## Results

### Respondent socio-demographic characteristics

A total of 22 state-level participants were interviewed from 10 states and the Federal Capital Territory (FCT). Respondents’ age ranged from 40 to 60 years. The majority 18(82%) were male while the median total duration of employment was 23.5 years. The respondents held leadership positions in SMoH and SPHCDA, with many being Deputy/Acting Directors 6 (27%) and Directors 5 (23%) and commissioners for health 2 (9%) (Table 1). Most 20 (91%) had been in their current position for at least 2 years.

**Table 1:** Background characteristics of participants

Socio-demographic characteristics	*n* %
Age (years)	
40–49	5 [23]
50–59	15(68)
60–69	2 [9]
Sex	
Male	18(82)
Female	4 [18]
**Duration in present employment (years)**	
< 1	5 [23]
1–5	14(64)
> 5	3 [14]
**Duration in present position (years)**	
< 20	8 [36]
20–30	8 [36]
30–40	6 [28]
**Position held**	
Deputy/Acting Director	6 [27]
Director	5 [23]
State Immunization Officer	4 [18]
Commissioner for health	2 [9]
Executive secretary SPHCDA*	2 [9]
Others	3 [14]
**Zone**	
NE	2 [9]
NW	2 [9]
NC	4 [18]
SE	4 [18]
SW	6 [28]
SS	4 [18]

*State Primary Health Care Development Agency

### Health services delivery volumes at the PHC during the COVID-19 pandemic

All participants acknowledged reduction in patients’ attendance at the PHCs while some also mentioned interruption in health services delivery. The decrease in facility utilization was more pronounced during the early stage of the pandemic particularly from March 2020 through June/July, 2020. Notably, there was a drastic reduction in the antenatal clinic attendance by pregnant women and the under-5 children outpatient visits across all regions of the country.

The movement restriction during lockdowns and the fear of contracting COVID-19 were the two most prominent reasons stated for reduction in health facility patients’ attendance. Where facilities were still in operation, fear of contracting the virus among patient and health workers was common in all regions.

On the supply side, some health workers did not go to work, while some facilities were instructed to close completely. The lockdown reduced the number of health workers who were able to commute to work especially those who did not have personal means of transportation. Some state governments (such as Lagos) tried to ameliorate this by providing ambulances that took frontline workers to work. Health workers were also given stickers to identify them as essential workers so that the law enforcement agents would allow them to move through the lockdown. Like the patients, the health workers were also scared of contracting COVID-19 infection and they encouraged patients that could be managed at home to stay away from the health centers. They also referred patients very readily to the next level of health care with minimal investigations. The decline in services were attributed majorly to the COVID-19 pandemic.

On the demand side, patients were unable to travel to health facilities because of lockdown restrictions. Participants also emphasized on the economic challenges and bank closures which reduced people’s ability to purchase goods and services including healthcare. Community members exhibited fears from the belief that COVID-19 was domiciled in the health facilities. People were further afraid of being isolated in the event that they were diagnosed with COVID-19.

### Differential impact of COVID-19 on LGAs

There was consensus between the participants from the different regions that urban areas had a higher burden of COVID-19 infection including disease incidence and case fatality. Consequently, there were more COVID-19 response activities in urban areas.

Participants in all regions believed that health service provision had returned to normal by June, 2022 especially for some suspended activities in the pandemic. Such activities included the integrated supportive supervision of health facilities which was believed to have returned to pre-COVID-19 levels. The isolation centers were no longer in existence and ad-hoc workers were no longer in employment. However, some COVID-19 prevention strategies such as the social mobilization, advocacy and risk communication were still on-going at the time of data collection.

### Challenges faced in maintaining essential health services

Key challenges were identified by participants. Human resources shortage was the most commonly mentioned challenge from 4 GPZs, 6 states (Lagos, FCT, Imo, Kano, Abia, Ogun) of the country. Other commonly mentioned challenges included: Shortages in the supply of Personal Protective Equipments (PPEs) 4 GPZs, 5 states (Imo, Lagos, Ogun, FCT, Gombe); fear of contracting COVID-19 among health workers 4 GPZs, 4 states (Imo, Ogun, FCT, Rivers); misconception, ignorance, socio-cultural issues 2 GPZs, 2 states (Rivers, Imo); lockdown/transportation 2 GPZs, 2 states (Abia, Lagos); and lack of equipment/waiting area 2 GPZs, 2 states (FCT, Oyo). Less commonly mentioned challenges included: training gap, inadequate referral, diversion of other facility budget lines to PPEs purchase, and insecurity. The challenges considered to pre-date COVID-19 included: human resources shortage, shortages in equipment and PPEs, poor infrastructure and inadequate funding.

The challenges faced in maintaining essential health services in different health systems pillars are highlighted below with sample quotes from individual respondents (Table 2):

**Table 2:** Challenges in maintaining essential health services in Nigeria: Themes, emerging subthemes and some quotes

Themes	Emerging subthemes	Examples of quotes
Leadership and governance	Accountability	*“….the challenge of diversion. of resources [budget for other facility needs]… for PPEs”* ***(NC)***
Finances	Funding availability	*“….insufficient funds has always been on ground. It is not really related to COVID-19. It has always been a case in most of the PHCs getting stipends to run the PHC like lightings, generators, pumping of water.”* ***(SW)***
Service delivery	Infrastructure	*“…majority of the health facilities do not have waiting area…”* ***(SW)***
	Accessibility	*“…referral, when somebody is positive having to evacuate from the hospital to the treatment centre, was a challenge”* ***(NC)***
	Acceptability	*“.misconceptions about the disease… with thoughts that there was no COVID-19 in the first place”* ***(SS)*** *“…person [patient], and/or…. relatives were not willing to adhere to the protocols,. what do you do?. socio-cultural issues,… where some people will say my pastor said.”* ***(SE)***
Human resources	Human resource shortage	*“The major challenge is… inadequate manpower which existed before COVID-19.”* ***(SW)*** *“Then during the pandemic too some health workers absconded…. Health workers who had the requisite capacity were quite few”* ***(SE)*** *“…there was some sort of shifting done to reduce the number of health workers working at the same time…”* ***(NC)*** *“They [workers] find it a bit difficult to get to their work place. Some of them have to use their workplace as home…”* ***(SW)*** .*It included even the health service providers. They were locked down. They could not even access the facilities”****(SE)*** *“Well, I will say the issue of the human resource for health; it has been a long-lasting challenge even before the pandemic. So, it was now heightened by the pandemic.”* ***(SE)***
	Health workers’ attitude	*“.even health workers were scared and they were not so committed to work because there was risk [of infection] now”* ***(NC)*** *“We had challenges with [the] attitude to work you understand? Some people were more reluctant”* ***(SS)***
Medicines and supply	Accessibility	*“.it was so bad that some doctors will even use their money to buy sanitizers and face masks so as to protect themselves”* ***(SW)*** *“.dearth in supply of PPEs… but that was at the initial period. Before COVID-19, there were no local manufacturers”* ***(SE)*** *“…challenges about the supply chain in terms of internal access to the PPE,… we put the PPE in the store and health workers in the emergency unit were not having access”* ***(NC)*** *“Rapid test equipment was not readily available at the beginning [of the pandemic]…”* ***(NC)*** *“…dearth in supply of PPEs was actually a challenge that was in existence beforehand”* ***(SW)***

### Leadership and governance

The respondent from the North Central (NC) zone explained that most of the resources allocated to various other activities in health facilities were redirected to meet the needs of COVID-19 response especially the provision of PPEs (Table 2):

…. the challenge of diversion… of resources [budget for other facility needs]… for PPEs.

### Finances

From the Southwest (SW) zone, a respondent stated that insufficient funding had always been a challenge in carrying activities such as providing electricity in the PHCs. The challenge pre-dated COVID-19 pandemic.

*“Insufficient funding has always been on ground. It is not really related to COVID-19. It has always been a case in most of the PHCs getting stipend to run the PHC like lightings, generators, pumping of water.” (****SW***,*)*

### Service delivery

The majority of the PHCs lacked infrastructure that could aid organization of services to provide physical distancing for the patients. A participant in the SW was quoted:

…majority of the health facilities do not have waiting area….

The health facilities experienced difficulty in transporting COVID-19 patients referred to isolation center for care. This was expressed by a participant in the NC zone:

*“…referral, when somebody is positive having to evacuate from the hospital to the treatment center was a challenge” (****NC****)*.

In the South-south (SS) and Southeast (SE) zone, the participants expressed concerns about patients’ misconceptions about COVID-19. Many patients did not believe that COVID-19 exist and as a result, were unwilling to adhere to facility COVID 19 prevention protocols. These misconceptions were reinforced by socio-cultural norms and reliance on dictates of religious leaders.

“.Misconceptions about the disease… with thoughts that there was no COVID-19 in the first place” (***SS****)*.

“…the person [patient], and/or.relatives are not willing to adhere to the protocols, …what do you do?.socio-cultural issues,…where some people will say my pastor said…” (***SE****)*.

### Human resources

Inadequate human resource which predated the COVID-19 pandemic was expressed by both northern and southern zone respondents across six states. However, this challenge was amplified by the pandemic. There was limited number of personnel with the requisite skills to perform tasks related to the response. The task shifting strategy implemented to share task and thereby, reduce the number of health workers in facilities at any one time, also reduced the human resource capacity in the PHCs.

*“The major challenge is… inadequate man power which has existed before COVID-19….” (****SW****)*.

*“Then during the pandemic too some health care workers absconded….health workers who had the requisite capacity were quite few” (****SE****)*.

*“….there was some sort of shifting done to reduce the number of health workers working at the same time…” (****NC****)*.

Respondents in SW and SE also described the challenges that health workers encountered in getting to the health facility during the early period of the pandemic due to the lockdown. This was said to compound the human resource shortages.

*“They [workers] find it a bit difficult to get to their work place some of them have to use their workplace as home …” (****SW****)*.

*“It included even the health service providers. They were locked down. They could not even access the facilities” (****SE)***.

The human resources shortages in the facilities was confirmed to have been a long-standing problem that existed before the pandemic across all regions of the country which was now amplified by the pandemic.

*“It [staff shortfalls] was on ground before …” (****SW****)*.

*“Yes, I said it that staff shortfall has been a long-term issue. The work is becoming voluminous everyday” (****SW****)*.

*“Of course, we have human resources gaps, before and even during the pandemic” (****NC****)*.

*“Well, I will say the issue of the human resource for health, it has been a long-lasting challenge even before the pandemic. So, it was now heightened by the pandemic…” (****SE****)*.

Health workers’ attitude to work was stated as being a challenge to utilization of PHCs by clients. Due to the fear of contracting COVID-19, health workers were not committed to work.

*“We had challenges with attitude to work you understand? Some people were more reluctant” (****SS****)*.

*“…health workers had a ground to be afraid because there were gaps [in] science” (****SE)***.

*“…even health workers were scared and they were not so committed to work because there was risk [of infection]” (****NC)***.

### Medicines and supplies

Respondents across most of the regions reported shortages in medical consumables such as PPEs, face masks and sanitizers especially at the beginning of the pandemic. One respondent decried challenges with the supply chain because of restricted access to PPEs even though some facilities had supplies locked up in the store.

*“… it was so bad that some doctors will even use their money to buy sanitizers and face masks so as to protect themselves”****(SW)***.

*“…dearth in supply of PPEs….but that was at the initial period. Before COVID − 19, there were no local manufacturers”****(SE)***.

*“…when we started there was really a challenge in the facilities because even face masks were running out. Sanitizers were running out because of the increased use.”****(NE)***.

*“…challenges about the supply chain in terms of internal access to the PPE. We put the PPE in the store and health workers in the emergency unit were not having access”****(NC)***.

*“Rapid test for SARS-CoV-2 was not available at the beginning [of the pandemic]” as* expressed by a respondent from the FCT **(NC)**.

The dearth in supply of consumables was confirmed to be a challenge that existed before the COVID-19 pandemic. However, the increase in the cost of some consumables such as PPE, gloves and face masks was a challenge that came with the COVID-19 pandemic.

*“Dearth in supply of PPEs was actually a challenge that was in existence beforehand”***(*SW)***.

### Mitigation strategies to health systems challenges during COVID-19 pandemic

Several interventions were implemented by state governments to address the challenges of maintaining essential health services (Table 3). State governments focused on the provision of consumables; recruitment, redeployment and provision of training for health workers; expansion of the infrastructural capacity; provision of vaccines, stipends, security and subsidizing health services costs. These interventions were in all regions of the country.

**Table 3:** Countermeasures to health systems challenges during COVID-19 pandemic in Nigeria: Themes, emerging subthemes and some quotes

Themes	Emerging subthemes	Examples of quotes
Leadership and governance	Political will	*“Government was ready to approve all the ongoing projects; all ongoing services, basic medical services were being provided. They also were fighting stigma within the facilities”(* ***NC)*** *“They [government] made some services affordable, available and accessible and within the reach of the community members. They were taking services even to the community outside the facilities, services like outreach services, information dissemination and empowerment.”* ***(NC)***
Finances	Funding availability	
Service delivery	Infrastructure	*“They [government] provided treatment centres for those who required admission”* ***(NC)*** *“Government built isolation centres all across the 20 local government in Ogun state that is the jurisdiction”* ***(SW)***
	Accessibility	*“For example, during COVID-19 pandemic women had emergency caesarean sections especially pregnant women. There is an ambulance that picks them and there is also another one that is called EMC[Emergency Maternal Care] services. It is a special service provided by the state government for maternal and new-born child free, up to this moment”* ***(NW)*** *“…The state government also provided ambulances, one ambulance to one local government. They gave ambulances and drivers. They also provided security…”* ***(SE)***
	Service prioritization	*“We know that personnel shortfall is a challenge, so of course, that is one of the reason why we did the prioritization, and we had to reprioritize some of our facilities”* ***(SS)*** *“During the pandemic we had shortfalls in the number of staff. There were gaps and the strategy we used was to either give appointments if it is not something [health condition] that is so serious; If it is not an emergency or you ask the patient to visit other health facilities that are not too far especially if it is a health facility that has been shut down because of COVID-19 and the services could not be accessed. To obtain the services that they required, we ask them to go to a nearby PHC, give them appointment so that they can access the needed health services…”* ***(SW)***
Human resources	Human resource shortage	*“They [government] gave allowances to adhoc workers for a few months, so the adhoc workers helped….”* ***(SE)*** *“…the recruitment [of health workers] has been in phases; some doctors have been recruited… we will recruit more nurses… and the community health extension workers…”* ***(SW)***
	Training	*“We tried to brief the health workers, organize workshops for them and tried to get them properly informed and well equipped by way of training so that they will be able to overcome some of the fear, you know lack of capacity brings fear…”*
	Motivation	*“Increasing the health workers hazard allowance is something that the government did….”* ***(SW)***
Medicines and supply	Accessibility	*“The state government provided PPEs, because there were also donations to them. Many private firms also donated and … they made it available for the public hospitals”* ***(SE)***

### Leadership and governance

Political will improved during the COVID-19 pandemic, state governments were positively disposed to improving health services delivery.

*“Government was ready to approve all the ongoing projects, all the ongoing services, basic medical services were being provided, they also were fighting stigma within the facilities”****(NC)***.

*“They [government] made some services affordable, available and accessible and within the reach of the community member. They were taking services even to the community outside the facilities, services like outreach services, information dissemination and empowerment.****” (NC)***.

Key interventions implemented across the regions were cascaded from state level to the LGA and facility levels down to the community. Across all regions, training and capacity building were stepped down to LGAs, facility heads and community. These activities were facilitated through LGA officers and community stakeholders.

*“Health worker training was also done for health workers at the primary care centres and the secondary facilities at each of the area council. So, all the activities, all the IPC was also done.” (****NC****)*.

*“At the state, we have a state officer, we have the Local Government officers, we also have the health facility officers. These trainings were cascaded down from the State to the Local Government and to the health facilities to ensure that the various layers of response are well equipped in terms of capacity.” (****SE****)*.

*“We train and monitor. We also conduct supportive supervision from the state level down to the local government levels then to the ward and facility level; we do that routinely. We check their knowledge gap and also do on the spot training for whichever gap that we are able to identify.” (****SE****)*.

*“…there were trainings that we received, training upon training which usually comes from the national to the State and then we step it down to the local government and then from the local government to the wards within local governments and the facilities.” (****SW****)*.

*“We work with the medical officers of health in the twenty-three LGAs and the heads of facility to redistribute our staffs.” (****SS****)*.

Coordination across levels of the health systems also ensured timely distribution of health facility materials:

*“The moment the supply comes into the state with immediate effect they write to the MOHs (Medical office of health) in the local government stating we have some materials for you, because we do not wait until the MOHs come to collect the materials, so we send a letter to them via email communicating the delivery time. E.g. we are bringing it tomorrow morning or we are bringing it this evening be available to receive it. The moment it gets to the MOHs, the MOHs step it down to all the facilities and PHCs with immediate effect.” (****SW****)*.

*“The state primary health board makes funds and logistics available at the local government level” (****SW****)*.

*“The intervention trickles down to the facility level. The State made sure that the issue of man power, issue of adequacy of jobs you know and consumables at the health facility are addressed at the highest decision level” (****SW****)*.

### Medicines and supplies

The COVID-19 response was supported by donor partners such as in the provision of PPEs. The government also mobilized funds from the private sector which was made available to the hospitals and PHCs.

*“The state government provided PPEs, because there were also donations to them, many private sectors also donated and… they made it available for the public hospitals” (****SE)***.

#### Service delivery

Regarding service delivery during the pandemic, interventions implemented included reorganizing service delivery for more facilities to render more services.

*“We had to reorganize our system to ensure that more facilities in some strategic locations were rendering more services, had more people to render services, you understand, 24/7. We actually had to do that” (****SS)***.

The government also built COVID-19 isolation and treatment centers to relieve the pressure on the hospitals and ensure COVID-19 patients had good care.

*“They* [state government] *provided treatment centers for those who required admission,” (****NC)***.

*“Government-built isolation centers all across the 20 local government in Ogun state that is the jurisdiction.” (****SW****)*.

In the Northwest (NW) zone, the government organized the Emergency Maternal and Child (EMC) services where they provided ambulances to pick up pregnant woman that required emergency surgery. Provision of ambulances was not limited to the NW region as other regions also mentioned government support by providing ambulances.

*“For example, during COVID-19 pandemic people there had emergency cesarean sections especially pregnant women. There is an ambulance that picks them and there is also another one that is called EMC services, it is a special service provided by the State government for Maternal and newborn child free up to this moment”****(NW)***.

Intervention strategies in facilities also included prioritization of facilities in terms of services and staffing needs, rescheduling of patients’ appointment that were not emergency cases.

Clients were also redirected from facilities that were shutdown to nearby facilities that could provide treatment services. Services prioritized included patient monitoring/treatment, immunization services and provision of ambulance for transportation.

*“Well, the patient monitoring evaluation and treatment were prioritized because we do not want to come down with a lot of mortality. So adequate equipment [and] consumables were provided by the State and the manpower involved were adequately remunerated and then the State paid a lot of money for them to maintain this service” (****SE****)*.

*“The services like maternal and child care…. those services are key. We want to make sure that mothers, pregnant mothers access care on time, the children too… Those that need to be immunized and all of that.” (****SW****)*.

*“The maternal, new born and child health services were prioritized and also the health workers themselves were prioritized because they are the frontliners” (****SE****)*.

*“…anybody that falls sick and gets to the hospital will receive care but we pay attention on pregnant women and little babies more because their own case is peculiar” (****SW****)*.

*“The mother too who attended antenatal clinic and even the test that will be run everything was done for free and was sponsored bby the PHC Board to the extent that they printed cards and gave it to them for free that they were not supposed to pay. The registration, everything was made free at that time. This is just to act as reliefs at that time for those who access health at the health facilities” (****SW****)*.

### Human resources

To address the shortfall in human resources, the SW region employed health worker cadres such as doctors and nurses in batches per time, as the budget could accommodate. In some other regions such as the SE, health workers were redeployed to work at facilities which were near where they lived to improve delivery. Ad hoc staff were also engaged to work for a few months.

Workshops were organized by the state governments to train and inform the health workers on IPC and to improve their skills. This helped to alleviate their fears on contracting the virus so as to alleviate their apprehension.

Health workers including adhoc staff were motivated by increasing the hazard allowance, which led to the increment in their monthly salary.

*“They [government] gave some allowances to adhoc workers for a few months. So those adhoc workers helped….The state government also provided ambulances, one ambulance to one local government. They gave ambulances and drivers…also provided security…” (****SE****)*.

*“Health care workers were also provided with the relief materials to also help them continue in their work” (****SE****)*.

*“Increasing the health workers hazard allowance is something that the government did….” (****SW****)*.

*“Yes, the government provided allowances to encourage those who were at the frontline to ensure that they [health workers] at least had something reasonable to hold on to while offering their services and apart from that government was coordinating the activities of the various fronts including that of security.” (****SE****)*.

*“Giving reliefs, packages, and giving us bonus that was all.” (****SW****)*.

*“Those that took part in surveillance were given certain stipends, those that did case management were given certain stipends, those that took part IPC, risk communication, point of entry was given certain stipends.” (****SS****)*.

*“Governor continued, was even giving transport stipends to surveillance officers, laboratory personnel, just to encourage them to do the work and so, these things were going on as a kind of stimulant, a kind of motivation to assist in getting the job done. So as at that period those things were not lacking for us, so that is what I can say about that*.” *(****SS****)*.

*“The support is the trainings that were done, stipends were paid adequately as at when due and the health workers were happy with that, as they carried out their duties” (****SE****)*.

*“The hazard allowance was increased, I think to about 15% or thereabout, so all those incentives were there for health workers to actually motivate them to do more, so the State government did that.” (****SW****)*.

*“Palliatives, all the health workers were given palliatives.” (****SW****)*.

*“All the health workers were given adequate and reasonable support; number one, in the FCT, they were well paid. Those that were directly involved [in COVID-19 control] were well paid by the honorable minister of the FCT, secondly, they were all provided at any given point in time with PPEs, they were also well trained to monitor patient, and even the family of those who died were given some support, I think some were promised land, I don’t know if they have given them. They were given high level of support.” (****NC****)*.

Other support granted by the State to motivate health workers included training, recruitment to support existing staff, provision of security, relief packages and ambulances.

*“I know I have talked about redistribution of workers, of course ad-hoc workers for those very few months, then some of the PPEs and some of the security, I think that’s the only thing I can say.” (****SE****)*.

*“To be sincere we have to appreciate the state government, at that time they even gave us accommodations, food and everything during the first pandemic. They support us with training of case management for us to take care of patients as well as series of other training. We all attended online training on oxygen therapy and it was even paid for” (****NW****)*.

*“At one point, it was difficult for health workers to move from one point to the other, so government aided the movement of health workers by providing certain things to identify them, also providing ambulances, movement support to enable them move from their homes. They also provided accommodation for health workers at the isolation center.” (****SE****)*.

*“And also, they bring in special teams to also support the teams on ground.” (****SE****)*.

*“Well, we did some form of reorganization and that did include the personnel. So, we had to increase the number of personnel in our focal facilities which increase the services” (****SS****)*.

*“Yes training has always been in existence so they do refresher training but during the COVID it become more intensified because of the session or season we are.” (****SW****)*.

#### Information systems

Respondents mentioned that government engaged in communication/sensitization programmes to improve service utilization using different media including the traditional and social media. The targets of the communication programmes were the community members including religious and ethnic groups. Communities, markets, churches and mosques were some of the places where the health promotion campaigns took place (Table 3).

#### Adaptations of the health systems during COVID-19

##### Sustainable adaptations

Table 4 shows the emerging themes on sustainable adaptations done by the health systems. Respondents considered the infection, prevention and control (IPC) infrastructure (taps for running water), the telehealth call center, the IPC protocols and the service reorganization, as sustainable. A respondent mentioned that each health facility had an IPC focal person and also IPC teams which the health system can continually optimize.

**Table 4:** Sustainable Health systems adaptations suggested by respondents

Themes	Emerging subthemes	Examples
Service delivery	IPC infrastructure	*“There are other interventions on personal hygiene, you know, we had taps, all the buildings, we built taps in front of a lot of our facilities, so we ensure that people still carry out personal hygiene and the health talks in relation to the facilities”* ***(SS)***
	Telehealth Infrastructure	*“.Then the call centre, you know we have the COVID-19 call centres and there is need to also allow it to continue beyond the pandemic to a general health call centre. That will be one of the legacies of the pandemic.”* ***(NC)***
	IPC protocols	“.*we keep on emphasizing and encouraging people to use it particularly in public places.”****(NC)*** *“.we should still use the PPEs and the donors, the partners should not still run away you know”* ***(SE)*** *“All the changes [IPC] we made, are changes that should continue to be used….”* ***(NC)***
	Service re-organization: IPC teams	“*…At every health facility, be it primary or secondary, there is an IPC focal person whom was not there before the pandemic. So, we now have an IPC focal person at the various health facilities that are now active.”****(NC)*** *“Yeah, okay so, the IPC teams are still in place, those ones are still in place. Of course, we will continue to do the training like you mentioned, yes of course, we will continue to do that”* ***(SS)***
	Service re-organization: task shifting	*“We will continue to ensure that our facilities carry out something that we said during the pandemic: peer mentoring, task shifting and task sharing.”*
Medicines and supplies	Provision of PPEs	*“Provision of PPEs should be done constantly on a monthly basis so that there is no time that you get there (health facilities) that you won’t see them [health workers] practicing these IPC….”* ***(SW)***
Human resources	Training on IPC	*“You know the IPC strategy gives training on personal hygiene, personal protection and all that, I think going forward, that should be enhanced…”* ***(SE)*** *“The IPC training structure was set up for each facility and network electronic platform set up to allow them collaborate is something that should be maintained because generally IPC is important not just for COVID-19 even for normal infection like diarrhoea. The IPC practices, I think is something that needs to continue.”* ***(NC)***
	Capacity building	*“…We had different types of training, training upon training during this period and then people are used to it and people are likely to continue doing it. it has been like a habit, a very good habit.”* ***(SW)***
Information	Public sensitization and community engagement	*“Sensitization has increased and has come to stay. Also, it has also helped in quick supply of the necessary materials by the health centers.”* ***(SW)*** *“And then the community engagement side for demand generation, we will also continue, it will not stop, so a lot of those groups that were formed as a result of COVID-19, they are still in existence, so we will still work with them to make sure that we use them for other interventions, going forward.”* ***(SS)***
	Virtual meetings	*“At the State level now, some of the changes that were made for example, if we want to do meeting now, it is not compulsory we do physical meeting…”* ***(SW)*** *“.there were more virtual meetings than during the pre-pandemic. It is something to hold to.”* ***(NC)***

Respondents considered that the training programs and capacity building efforts (especially the ‘network electronic platform’), implemented during the pandemic were sustainable. They opined that IPC training should be mainstreamed because the topic was broad and had impact on prevention of other infectious disease areas apart from COVID-19.

Respondents also mentioned that the volunteer groups formed during the pandemic for community sensitization and community engagement, were retained and would be used for other intervention programmes. Health teams have also retained the virtual mode of conducting team meetings.

### Unsustainable adaptations

Respondents considered some adaptations in financing, service delivery and supplies, as unsustainable (Table 5). The funds that the government mobilized in form of incentives to health workers, stipends for campaigns team members and payment for other ad hoc staff such as town criers, were no longer being provided. The free testing and healthcare for COVID-19 patients which governments implemented was not sustained. The health workers who were redeployed have returned to their pre-pandemic assignments. In addition, all the services rendered to patients at the COVID-19 treatment centers including treatment, accommodation, consumables, were free and therefore, considered unsustainable. This also included the free consumables supplied to the health workers.

**Table 5:** Unsustainable Health systems adaptations suggested by respondents

Themes	Sub-themes	Examples
Financing	Provision of funds, stipends for health workers and payment for campaigns	*“Well, you know during that period, State governments provided enough funds for health care workers and since the pandemic has subsided, those funds are not being provided…”* ***(SE)*** *“Well, for me the way I look at it, it is more of funding. You know it is capital intensive, so getting the funding is one of the biggest things, that I think the government can do,”* ***(NC)*** *“Even funding for some of the social media campaigns and all that, not all of them are sustainable, you understand? To be paying the town announcers regularly to go into the communities and all, no, not all are sustainable”* ***(SS)*** *“The changes that are unsustainable are directly linked with sustainable funding, now funding is no longer sustainable because of fatigue. …,.”* ***(NW)***
Service delivery	Free healthcare and COVID-19 testing	*“Giving health for free, ante natal all these cards that were done [Free health care for example, antenatal care]. In fact, I am not even sure before the end of pandemic that we will be able to sustain it all through I am not sure”* ***(SW)*** *“The only thing that is not sustainable that I can think of right now is, you know if I tell you go and ensure that you stay at that border, anybody that passed there, test them, it is not sustainable because they don’t test people again at the border, so it’s not sustainable.”* ***(SS)*** “*So that continuous free testing may not be sustainable. I think that it’s a very good strategy to remove the load from the government by setting up these private laboratory consortiums”****(NC)***
	Reshuffling and redistribution of staff	*“The reshuffling of staff, redistribution of staff, [is] not sustainable. You know, it’s a process. we are not just doing it at once but we are getting staffs to go back to the different facilities that were not prioritized at that point in time.”* ***(SS)***
	COVID-19 treatment centres	*“If you talk of changes that were made that are unsustainable of course they would include creation and establishment of treatment centres. For those who were infected, there were a lot of intensive or rather serious expenditure that was required to keep people in that place, for example, every patient that was on treatment, everything was free from the lowest things they eat, from food to medication, providing them with accommodation, providing isolation, providing all the machineries, PPEs that all the people that managed them used, all those things are not sustainable.”* ***(NC)***
Medicine and supplies	Free consumables e.g. facemasks, PPEs	*“It requires high resources for people to be using mask, buying it daily and using it. It is not economically wise and is not sustainable. Then, traditionally or culturally also people are not used to this kind of life”* ***(NC)***

## Discussion

### Summary of findings

The qualitative study selected senior persons in decision-making positions. Respondents acknowledged a reduction in patients’ attendance at the PHCs and interruption in service delivery. This prominently affected antenatal care attendance by pregnant women and the care for the under-5 children across all regions in the country. There was consensus among the regions that the urban communities had a higher burden of COVID-19 infection making the activities around COVID-19 control more intense in these communities. Unfortunately, this negatively impacted the provision of care in health facilities in these communities, leading to a negative impact on provision of EHS.

The challenges experienced in maintaining essential health services cut across the pillars of the health systems. Resources were reallocated to COVID-19 control activities from other budgetary lines due to insufficient funds to implement control activities. The infrastructure of most of the PHCs could not accommodate changes in service reorganization which was needed to enable physical distancing. It was also challenging to transport referred COVID-19 patients to isolation centers. Patients had misconceptions on the cause and transmission of COVID-19 and were unwilling to adhere to facility protocols. There was severe shortage of human resources which predated and was accentuated by COVID-19 control interventions such as lockdowns, staff redeployment and task shifting. Health workers were reluctant to discharge their duties because of fear of contracting the infection. There was inadequate consumables for use albeit sometimes due to deficient supply chain management.

Several mitigation strategies were implemented to address the challenges encountered. Political will towards improvement of health service projects was increased during the COVID-19 pandemic. This was reflected in government efforts to make health services available, accessible and affordable. Efforts were also made to provide consumables, recruit both permanent and ad-hoc staff, motivate existing health workforce, and redeploy/train health workers. The health infrastructure capacity was also expanded across regions, to free up spaces for provision of EHS by building/renovating COVID-19 isolation and treatment centers. Service delivery was also reorganized by rescheduling appointment for non-emergency to a later date and prioritizing essential services such as immunization, maternal and child care. Health promotion campaigns to groups and communities, were conducted to improve service patronage. Sustainable systems adaptations included IPC and telehealth infrastructure, IPC protocols, IPC teams and focal persons, training and capacity building, virtual meetings and community groups set up for sensitization and engagement. Unsustainable adaptations included funding, free healthcare and consumables, redistribution of staff, and the maintenance of COVID-19 treatment centers.

### Results in the context of the literature

The COVID-19 pandemic disrupted EHS in almost all countries of the world and the disruption continued for over two years in more than 90% of countries surveyed by the WHO [30]. Particularly affected were the maternal and childcare services as corroborated in both quantitative and mixed methods design studies [31–33]. Our study corroborated findings from surveys among health workers and community members in Burkina Faso, Ethiopia and Nigeria, confirmed partial-to-total interruptions in health services delivery and utilization especially maternal and child health services [34] due to lockdowns, fear of infection/stigmatization, misconceptions/misinformation about the disease, stockout of drugs, and lack of transportation due to lockdowns [35, 36]. As noted in this study, the disruption affected most services to the extent that some PHCs with low capacity were closed down. Studies indicated that disruptions appeared to affect disproportionately maternal and child care including immunization [30]. As noted in the WHO survey and as corroborated by our study, the major barriers to health service recovery were health systems challenges which predated the COVID-19 pandemic. Very prominent pre-existing health systems deficiencies identified by our study were in the human resources, service delivery and the finance pillars.

The adaptations to service delivery implemented in healthcare facilities were similar across regions in Nigeria and notably, were designed to reduce patient inflow. Non-emergency cases were discouraged from accessing clinics and follow-up appointments were rescheduled because the facilities lacked the capacity to implement the recommended physical distancing between patients. In Ghana [37] similar adaptations were made to routine healthcare service delivery which also aimed at reducing patient flow to the health facilities. In this study, only clients with extremely important conditions were encouraged to visit the health facilities, appointments were reduced, non-essential medical and surgical procedures were less prioritized.

Although, facility closures occurred in most settings around the world during lockdowns because there was no health manpower to provide services [3], the telemedicine infrastructure which existed before the pandemic in some settings, were deployed to bridge the gap in consultation demands [3, 38]. Nigeria developed a telehealth call center which was mainly for COVID-19 case finding but provides opportunities for general health consultations use.

Also, some health professionals were reassigned to COVID-19 control programmes which ultimately affected services such as home visits, immunization and other community health services [37]. A study conducted in Lagos, Nigeria highlighted the willingness of community health workers to function as care providers during the pandemic but were challenged by heavy workload and lack of transportation [39]. These recommendations informed some of the decisions to improve health workforce care packages including financial incentives and employment of additional staff [39]. 

Limited evidence exist in the literature on the challenges encountered in maintaining EHS in health systems. In Bangladesh, similar challenges were reported as we found in our study. The demand pull challenges in Bangladesh included fear of COVID-19 infection, difficulty with commuting during lockdown and reduction in health seeking behavior emanating from closure of health facilities without providing alternatives [40]. Also, as found in our study, health resources were redirected to COVID-19 leaving other important health programmes deprived. Likewise, there were staff shortages which predated COVID-19: Acting in synergy with panic among health workers, more health facilities and programmes were further abandoned as similarly documented in our study.

The literature was richer in terms of mitigation and adaptation strategies implemented to maintain EHS during the COVID-19 pandemic. Kabwama et al., used the same health systems pillar thematic framework to analyze the interventions implemented in maintaining EHS in Uganda [41]. Prominent in the Uganda analysis was the private sector engagement for public-private partnership in fund mobilization as reported in our Nigeria analysis. Unique adaptation in service provisions in Uganda involved leveraging patient networks to deliver medicine which was not found in our analysis. The Ugandan study appeared to focus more on general interventions that were not specifically directed at challenges in maintaining EHS contrary to what our study did. The mitigation strategies implemented in Bangladesh closely mirrored what our study found such as provision of consumables under the medicines and supply pillar, fund mobilization under the leadership/governance and finances pillars among others [40].

Perhaps, the most robust survey on service adaptations involved 129 countries and was conducted by the WHO [30]. It was clear that in all countries, services were shifted off the health facilities and moved to home-based or to tele-infrastructure. Low and middle income countries like Nigeria may benefit from such easily adaptable strategies because creating separate facilities for COVID-19 and EHS delayed implementation as a result of the considerable financial investment required. Policy makers involved in emergency and epidemic preparedness plans may incorporate proactive plans to achieve rapid implementation of similar strategies. Other prominent cross-cutting mitigation strategies reported across countries in the WHO survey included healthcare financing, health workforce training and capacity building, procuring of essential medicines and consumables, risk communications and community engagement.

### Implication of findings and lessons learned

The WHO recommends that advanced planning and long-term investments in health systems is important for epidemic preparedness and in safeguarding the continued provision of EHS during a health crisis [42]. Findings derived from this study are imperative for a robust epidemic preparedness plan. Strategies to maintain supply and demand for EHS should be incorporated as essential elements of epidemic preparedness plans. Response to health crisis require a more holistic and proactive approach at planning. The challenges facing the Nigerian health system are long-term which will require considerable and consistent efforts to resolve. Thus, learnings on mitigation strategies and adaptations during the COVID-19 pandemic would be applicable for future public health emergencies as well as routine health services delivery. The sustainable adaptations can potentially serve as a foundation for a gradual, planned, and intentional investments in the core functions of the Nigerian health system in order to improve its resilience and preparedness. For example, maintaining a pool of potential ad hoc volunteers consisting of retired health workers and community volunteers who can be mobilized at short notice. Also, the partnership built during the COVID-19 pandemic between the government of Nigeria and the private sector could be strengthened and optimized for epidemic preparedness and EHS delivery. The government at all levels received funds and donations from the private sector which was channelled to COVID-19 control and health care service delivery.

Our study also highlights the importance of adequate and timely public health messaging. Misconceptions and misinformation were rife during the COVID-19 pandemic in Nigeria [23]. Also noted [23], most of the information provided were technical and focused on prevention of COVID-19, with only minimal messaging on the provision/utilization of EHS. Thus, on the social media, misconceptions festered and was a major cause of demand-pull decline in EHS utilization by communities. Both patients in need of treatment and those who were on follow-up appointments, largely stayed away from the health facilities due to fear of contracting COVID-19. Health facilities were stigmatized, and health providers discriminated against for fear of contracting the virus. Another driver of decline in demand was the fear of testing positive and being isolated [43]. Although, adaptations to EHS later reduced the need for physical contact with the health facilities, a large proportion of potential clients stayed away from the formal health system. The learnings derived from adaptations during the pandemic could provide opportunities for a transformative evolution of the primary health care system in Nigeria. Before the pandemic, across the country, only about 20% of the PHCs were assessed as functional [28], resulting in consultation overload of the secondary and tertiary facilities. The participants considered the telehealth call center to be a sustainable innovation. The Nigerian health system could benefit from upgrading and expanding telemedicine infrastructure to shift some of the PHC overload to this platform. This will enhance an elastic, epidemic prepared EHS delivery system.

As confirmed in this study, poor funding was a systemic challenge that predated COVID-19 pandemic. EHS delivery suffered major set-backs partly because the meagre financial resources available for healthcare delivery were diverted to COVID-19 control. The budget for the State PHC Board in a state in North Central zone of Nigeria was reduced by 11.5% in order to secure funds for COVID-19 control activities [44]. The government was able to raise some funds mainly from the private sector most of which was deployed towards public health measures for COVID-19 control with little investment to strengthen the health system [45]. Public-private partnership could be strengthened to form an extra-budgetary sovereign wealth fund which will be used for emergency health purpose only and which can be mobilized at short notice. The state governments demonstrated commitment to long-term public health investments and reforms during, and in the immediate post-pandemic period [46]. A sustained commitment will improve the overall performance of primary healthcare in Nigeria in the near future.

#### Strengths and limitations of the study

The strength of this study is that participants were actors at the sub-national (state) -level. They were senior personnel who were decision makers in COVID-19 control and provision of EHS. They had good knowledge of activities that transpired in the states during the COVID-19 pandemic. Also, we sampled participants from all geopolitical zones of Nigeria in the interviews, which ensured representativeness. We translated and back-translated tools across zones to ensure accuracy.

The tool was designed using the conceptual framework developed by Kruk et al. [16], which was not initially based on the health systems pillars. It is possible that data on some health systems pillars exist which were not captured during the interviews. Conceptual framework used in the Kruk’s framework are not strictly health systems pillars or building blocks. Our study recruited mainly senior personnel in the ministries which might skew observations without the views of the junior personnel. Readers should interpret the findings with the view that potential richer health systems context may exist.

## Conclusion

This study showed that there were significant challenges in maintaining essential health services delivery and utilization during the COVID-19 pandemic in Nigeria. The maternal and child care services were particularly affected. The core health systems challenges which prevented the maintenance of EHS delivery were mainly in the human resources, service delivery and the financing pillars. The mitigation strategies and adaptations implemented were important contributors to EHS recovery especially in the high resilience LGAs and have implications for future epidemic preparedness plans.

### Electronic supplementary material

Below is the link to the electronic supplementary material.

Supplementary Material 1

## Data Availability

The datasets used and/or analysed during the current study are available from the corresponding author on reasonable request.

## Abbreviations

**ANC**: Antenatal Care
**EHS**: Essential Health Services
**FMoH**: Federal Ministry of Health
**FCT**: Federal Capital Territory
**GPZs**: Geopolitical Zones
**HR**: High Resilience
**IPC**: Infection Prevention and Control
**LGA**: Local Government Area
**NC**: North Central
**NE**: North East
**NHMIS**: National Health Management Information System
**MNCH**: Maternal, Neonatal and Child Health
**NW**: North West
**PHC**: Primary Health Care
**PHCs**: Primary Health Centres
**PPE**: Personal Protective Equipment
**SE**: South East
**SMoH**: State Ministry of Health
**SPHCDA**: State Primary Health Care Development Agency
**SS**: South South
**SW**: South West
**WHO**: World Health Organization
