# Supplementary material for: Health systems challenges, mitigation strategies and adaptations to maintain essential health services during the COVID-19 pandemic: learnings from the six geopolitical regions in Nigeria
Source: BMC Health Serv Res. 2024 May 14;24:625. doi: 10.1186/s12913-024-11072-2 (PMC11092264; doi:10.1186/s12913-024-11072-2)
Supplement: Supplementary file 1 — Supplementary Material 1 [file 12913_2024_11072_MOESM1_ESM.docx]

**Key informant interview (KII) guide – State Respondents**

*[Note: If time is limited,* ***bolded*** *questions should be prioritized].*

*Hello, my name is {NAME} from Hanovia Limited. We are conducting a study on behalf of Global Financing Facility in collaboration with University of Ibadan (UI). This study is aimed at understanding how Nigeria’s health system responded and adapted to the COVID-19 pandemic and to learn more about how primary health care centers provided routine, essential health services during the COVID-19 pandemic in your state.*

*Your State has been randomly selected to participate in the study and as a stakeholder at the State level, you have been selected to participate in the study. So, I am here to interview you so that we can learn from your experience on the subject matter mentioned earlier. As a result, I will ask you some questions which i would like you to provide answers to as best as you can. We hope that your answers to these questions will help improve emergency preparedness and response especially in maintaining essential health services during a pandemic and to guide the government, stakeholders and other partners in the development of plans that would address these challenges in Nigeria.*

*The discussion will last between 45 to 60 minutes. We would like to ask for your permission to record the discussion so that we can correctly capture everything that would be discussed. Please feel free to tell us your opinion and note that all these discussions remain very confidential, while your name will not be mentioned in any way when the report is being written. (USE THE CONSENT FORM)*

*Do you have any questions so far? 1. Yes 2. No*

*Do I have your permission to proceed? 1. Yes 2. No*

*Signature/Thumbprint of Participant___________________ Date:____________________*

*(Interviewer should record the following in the notebook and tape recorder).*

*Biographical details- please capture:*

Interviewer Code: /_______/

- Age
- Sex
- Duration of employment
- Duration in present position
- Position/role
- Type of Facility
- StateDate of Interview
- Start time: /__/___/___/___/ Time Completed: /__/___/___/___/

**Context on the study participant and their role**

1. Please **briefly** describe your involvement in response to the COVID-19 pandemic in your State

*Probe: for general and specific measures taken in responding to COVID-19 during the pandemic*

1. In what specific ways have your job responsibilities changed because of the COVID-19 pandemic?

*Probe: to get more ways that the respondent’s job responsibilities has changed due to COVID-19 Pandemic*

**Services used during COVID-19**

*We are interested in how facilities continued to provide essential services, like maternal, newborn and child health services, during the pandemic. Now, we are going to ask you a few questions about these services and how they were impacted by COVID-19.*

1. Across your state, can you compare patient attendance at primary health care centers during the pandemic (from March 2020 until now) to the period before the pandemic?
   1. Was the change in patients’ patronage more noticeable during certain periods of the pandemic? If yes, what periods of the pandemic?
   2. Was the change more noticeable among certain patients?
      1. *Probe specifically for pregnant women and children under-5 years.*
   3. What were the reasons for the change in patient attendance?
      1. *Probe for reasons for changes in service use by demand side (e.g. patients afraid, lack of money for treatment due to job changes, travel restrictions/lockdowns)*
      2. *Probe for change in service due to supply side* *(e.g. couldn’t treat patients, had to close down clinics, refer patients out)*
   4. Are the changes in number of services provided more influenced by COVID-19 or other issues?
      1. if Yes, please explain
      2. If other issues, describe.
   5. Please explain any differences in how different LGAs in your state have been impacted by the pandemic.
      1. What are the impacts?
      2. If certain LGAs, describe.
   6. Do you feel like things have changed to “normal” in your state as compared to during the Covid-19 pandemic?? Why or why not?

*Probe: for essential health services*

**Monitoring/data use**

1. Describe how the state monitors COVID-19 trends such as the number of cases in the state? *(Probe: for testing facility, documentation, reporting, etc.)*
   1. How did this monitoring vary across LGAs in your state?

**Self-regulation**

1. **What are the challenges that facilities in your state faced in maintaining routine services during the pandemic?**

**Ask 5a for all the stated challenges**

1. Tell me more about the challenge you identified. Is that a new challenge since the pandemic started in March 2020, or is it a challenge you had before the pandemic started?

*i. Probe for longstanding challenges (e.g. staffing shortfalls, funding shortfalls that pre-dated the pandemic, delay in fund release, equipment, socio-cultural issues) versus new challenges (e.g. supply chain disruption, PPE needs)*

1. **Identify the specific interventions the state government took to help overcome those challenges.**
   - 1. Were any specific services prioritized by the state for support?
     2. How were these interventions stepped down to the LGAs?
     3. How were these interventions stepped down to the facilities?
     4. Which organizations or individuals helped to overcome the challenge?
     5. Please share any challenges you had implementing these interventions at the local level.
     6. Were new strategies developed by the state to overcome the challenge(s) mentioned?
     7. Were there any factors that prevented those new strategies developed by the State from yielding results?

**Adaptive – short term**

1. **Describe the activities that encouraged people to continue to come or prevented them from coming to the primary health care center in the state.**
   1. How did the state government work with communities during the pandemic?
      1. Who did you work with and why?
         1. *Probe for specific actors at the community level*
      2. How did these approaches change over time? *(e.g community engagement, community sensitization/mobilization)*
      3. Do you think these approaches increased service use? What were the challenges faced? What were the successes you had in your state?
         1. *Probe for contextual considerations like preexisting relationships with community actors, or challenges like a lack of pre-existing outreach services*
         2. *Probe for specific barriers that prevented attendance and were not captured previously*
   2. Narrate how your state provided support for health workers during the pandemic.
      1. *Probe for task-shifting, upskilling of workforce required for the maintenance of essential health services, and workforce strategy in balancing pandemic response vs. EHS maintenance*
      2. Was this support new during the COVID-19 pandemic, or did it exist before the COVID-19 pandemic?

**Integrated capacities / planning**

1. Explain any actions the national government took to help facilities in your state maintain services during the pandemic.
   1. Probe for:
      1. How were these actions communicated from the national to the state level?
      2. Which of them was the most impactful?
      3. What were the challenges encountered in implementing these actions?
   2. Describe any changes made to these national actions based on the context of your state.
2. Identify changes made in the way state and local government agencies worked together during the pandemic.
   - 1. Who was in charge of developing these changes?
     2. Who was in charge of Implementing these changes?
3. **Can you share any peculiarities we should know about “X” LGA in terms of essential health services delivery?**
   1. *Probe for contextual factors before the pandemic like previous health systems strengths, funding, partners, etc.*
   2. *Probe for pandemic-specific factors*

**Relevance to MNCH**

*We are particularly interested in how (LGAs, facilities, communities) maintained maternal, newborn, and child health services during COVID-19. (if needed) For example, skilled birth attendance, ANC, routine child immunizations, and overall outpatient consultations.*

1. How did the COVID-19 pandemic change delivery of maternal, newborn, and child health services in primary health care centers specifically?
   1. *Probe for any differences between these services* (*skilled birth attendance, ANC, routine immunizations, and overall outpatient consultations)* *and others*
2. What specific things, if any, did your state do to make sure these services were available?
   1. *Probe for differences in general adaptations versus specific changes for MNCH services*

**Adaptive – long term**

*Thank you for sharing these experiences, it is very helpful for us. We are almost done with our questions. Finally, we want to learn about how the lessons learned during the pandemic might influence the future.*

1. **Share the changes you made during the pandemic that you think you will keep using when the COVID-19 cases subside** *(for example, you might have provided new infection prevention and control trainings to health care workers that you want to continue.)*
   1. Explain the changes you made during the pandemic that are unsustainable.
   2. Tell me more about why they are or were unsustainable.
   3. Share the lessons learnt that you would like to share with other States**.**

**Snowball sampling – for data collector insight and/or follow up work**

1. **Tell me anyone else you think we should talk to in your state.**
2. **We are also collecting data in X LGAs; tell me anyone specific there that we should speak to.**

*Thank you for your time. This is the end of our questions.*

*Do you have anything else you would like to share with us?*

*Do you have any questions for us?*

*Thank you again. We look forward to sharing the results with you once we have completed the study. If you have any questions for us after we leave, you can reach us at:*

*[*[*hhassanu@hanovialimited.com*](mailto:hhassanu@hanovialimited.com) *or 07045079180]*
